# Fetal Growth Restriction Alters Cerebellar Development in Fetal and Neonatal Sheep

**DOI:** 10.3389/fphys.2019.00560

**Published:** 2019-05-22

**Authors:** Tamara Yawno, Amy E. Sutherland, Yen Pham, Margie Castillo-Melendez, Graham Jenkin, Suzanne L. Miller

**Affiliations:** ^1^ The Ritchie Centre, Hudson Institute of Medical Research, Monash University, Clayton, VIC, Australia; ^2^ Department of Obstetrics and Gynaecology, Monash University, Clayton, VIC, Australia

**Keywords:** cerebellum, preterm, term, fetal sheep, fetal growth restriction, brain injury, blood-brain barrier

## Abstract

Fetal growth restriction (FGR) complicates 5–10% of pregnancies and is associated with increased risks of perinatal morbidity and mortality. The development of cerebellar neuropathology *in utero*, in response to chronic fetal hypoxia, and over the period of high risk for preterm birth, has not been previously studied. Therefore, the objective of this study was to examine the effects of FGR induced by placental insufficiency on cerebellar development at three timepoints in ovine fetal and neonatal development: (1) 115 days gestational age (d GA), (2) 124 d GA, and (3) 1-day-old postnatal age. We induced FGR *via* single umbilical artery ligation (SUAL) at ~105 d GA in fetal sheep, term is ~147 d GA. Animals were sacrificed at 115 d GA, 124 d GA, and 1-day-old postnatal age; fetuses and lambs were weighed and the cerebellum collected for histopathology. FGR lambs demonstrated neuropathology within the cerebellum after birth, with a significant, ~18% decrease in the number of granule cell bodies (NeuN+ immunoreactivity) within the internal granular layer (IGL) and an ~80% reduction in neuronal extension and branching (MAP+ immunoreactivity) within the molecular layer (ML). Oxidative stress (8-OHdG+ immunoreactivity) was significantly higher in FGR lambs within the ML and the white matter (WM) compared to control lambs. The structural integrity of neurons was already aberrant in the FGR cerebellum at 115 d GA, and by 124 d GA, inflammatory cells (Iba-1+ immunoreactivity) were significantly upregulated and the blood-brain barrier (BBB) was compromised (Pearls, albumin, and GFAP+ immunoreactivity). We confirm that cerebellar injuries develop antenatally in FGR, and therefore, interventions to prevent long-term motor and coordination deficits should be implemented either antenatally or perinatally, thereby targeting neuroinflammatory and oxidative stress pathways.

## Introduction

Fetal growth restriction (FGR) complicates 5–10% of pregnancies and is associated with increased risks of perinatal morbidity and mortality (Bernstein et al., 2000). FGR is linked to adverse perinatal outcomes, including stillbirth, prematurity, and need for neonatal intensive care; as well as long-term adverse outcomes including metabolic syndromes, hypertension, and neurological deficits (Baschat, 2011; Mayer and Joseph, 2013; Albu et al., 2014; Drost et al., 2018). FGR is principally caused by placental insufficiency, which results in reduced utero-placental transfer of oxygen and nutrients to the developing fetus (Figueras and Gardosi, 2011). FGR can be induced experimentally to examine the neuropathology associated with FGR (Swanson and David, 2015). In sheep, restricted placental growth and function can be induced by surgical removal of the major endometrial caruncles from sheep before mating, causing a 31% reduction in body weight (Robinson et al., 1979; Harding et al., 1985). In rodents, uterine artery ligation models have been widely described in pregnant rats, guinea pigs, and mice (Basilious et al., 2015). Regardless of the experimental model, the fetus responds to chronic hypoxic challenge with a redistribution of cardiac output to favor the brain – termed brain sparing – but this does not ensure normal brain development (Miller et al., 2016). Chronic hypoxia results in widespread cerebral cellular and axonal lipid peroxidation, and white matter (WM) hypomyelination and axonal damage (Miller et al., 2014). FGR in lambs also induces cerebrovascular changes in the WM and causes disruption to the blood-brain barrier (BBB) (Castillo-Melendez et al., 2017). Developmental delays in motor and behavioral function are also present soon after birth in FGR lambs (Miller et al., 2014). These may be attributed to mal-development of the motor and coordination center within the brain, the cerebellum (Volpe, 2009). However, the *in utero* effects of placental insufficiency, chronic hypoxia, and FGR on cerebellar structure and development are not well understood.

The cerebellum is one of the first brain structures to differentiate, but one of the last to mature (Triulzi et al., 2005). In the last trimester of human gestation, and often coinciding with preterm birth in FGR offspring, cerebellar development undergoes a phase of rapid growth and interconnection with other cerebral structures (Volpe, 2009), which then continues into the first postnatal year (Abraham et al., 2001). Therefore, chronic hypoxia and/or preterm birth in the last pregnancy trimester may adversely impact cerebellar maturation and may also alter connectivity and subsequent development of other brain regions (Limperopoulos et al., 2010). The development of the cerebellum and cerebellar morphology has been described in fetal sheep (Rees et al., 1988, 1997). Purkinje cells are present by 100 days gestational age (d GA; term is 145–148 d GA) and branching of their dendritic tree is attained by 140 d GA. However, the presence of immature and mature dendritic neuronal markers indicates that considerable remodeling of Purkinje cell branching is still occurring in late gestation. Rat and guinea pig models of FGR show altered postnatal development of the cerebellum, including deficits in neuronal and glial formation (Mallard et al., 2000; So et al., 2013; McDougall et al., 2017). The ontogeny of cerebellar neuropathology *in utero*, in response to placental insufficiency and chronic fetal hypoxia, and over the period of high risk for preterm birth, has not been previously studied.

Therefore, the objective of this study was to examine the effects of FGR induced by placental insufficiency on cerebellar development. We induced late-onset FGR in fetal sheep *via* single umbilical artery ligation, which causes chronic fetal hypoxia and hypoglycemia but does not routinely cause preterm birth, as per late-onset FGR in the human (Baschat, 2014). We examined the cerebellum at three timepoints in ovine fetal and neonatal development; aim: (1) 115 d GA, (2) 124 d GA, and (3) 1-day-old postnatal age. Although the ontogeny of sheep cerebellar development is not well characterized with respect to human cerebellar development, we selected these timepoints to broadly correspond to human brain cerebrum and cerebellar development from 28 weeks through to infancy in the human (Rees et al., 1997; Back et al., 2012).

## Materials and Methods

### Animal Surgery

The surgical and experimental procedures undertaken in this project were approved by Monash Medical Centre Animal Ethics Committee A (MMCA2010/03). Surgery was performed on 26 Border-Leicester-cross pregnant ewes carrying twins (Aims 1 and 2) or a single fetus (Aim 3; Table 1) to induce late-onset FGR *via* SUAL, as previously described (Miller et al., 2012; Alves de Alencar Rocha et al., 2017). Briefly, anesthetized ewes underwent surgery to instrument each fetus with a femoral artery catheter and an amniotic catheter for determination of blood gases and to administer antibiotics, respectively. The SUAL procedure was performed by ligating one of the umbilical arteries with two tight silk ligatures (FGR) and, in the sham procedure, the umbilical cord was exposed but not ligated. SUAL induces necrosis of approximately half of the placental cotyledons, thereby resulting in chronic placental insufficiency with reduced transfer of oxygen and glucose to the developing fetus (Miller et al., 2014). A maternal jugular vein catheter was inserted for antibiotic administration prior to recovery of the ewe.

**Table 1:** Fetal and neonatal outcomes.

	115 d GA		124 d GA		1-day-old lamb	
	Control	FGR	Control	FGR	Control	FGR
Number of ewes	6		5		8	7
Number of fetuses	6	6	5	5	8	7
GA at surgery	107 ± 1.0	107 ± 1.0	104 ± 0.6	104 ± 0.8	105 ± 1.2	105 ± 0.9
Male:female	4:2	4:2	2:3	2:3	4:4	3:4
Body weight (kg)	1.9 ± 0.2	1.6 ± 0.1	3.5 ± 0.1	2.5 ± 0.2^*^	4.8 ± 0.5	3.5 ± 0.5^*^
Brain weight (g)	34.3 ± 1.6	34.4 ± 1.9	48.3 ± 2.0	43.9 ± 1.6	55.6 ± 2.7	51.3 ± 2.7
Brain:body weight (g/kg)	18.1 ± 1.3	21.8 ± 2.2	13.8 ± 0.5	17.0 ± 1.1^*^	11.8 ± 0.4	16.1 ± 1.8^*^
**Blood gas parameters (average across gestation)**						
Arterial oxygen saturation (%)	59 ± 1.3	52 ± 1.3^*^	70 ± 0.8	58 ± 0.8^*^	62 ± 1.5	43 ± 2.0^*^
**Cerebellum**						
Cross-sectional area (mm^2^)	1.3 × 10^5^ ± 1.2 × 10^4^	1.3 × 10^5^ ± 1.1 × 10^4^	1.6 × 10^5^ ± 1.6 × 10^4^	1.8 × 10^5^ ± 1.6 × 10^4^	2.1 × 10^5^ ± 1.9 × 10^4^	2.2 × 10^5^ ± 1.3 × 10^4^
Cross-sectional area:body weight (mm^2^/kg)	7.4 × 10^4^ ± 1.0 × 10^4^	8.1 × 10^4^ ± 6.0 × 10^3^	4.7 × 10^4^ ± 4.7 × 10^3^	7.1 × 10^4^ ± 1.0 × 10^4^	4.7 × 10^4^ ± 4.7 × 10^3^	6.2 × 10^4^ ± 4.3 × 10^3^ ^*^
EGL width (Mm)	22.0 ± 1.6	29.6 ± 2.6^*^	21.7 ± 2.3	21.5 ± 3.3	14.1 ± 0.7	14.0 ± 0.9
ML width (mm)	142 ± 6	112 ± 5^*^	160 ± 12	150 ± 11	173 ± 8	195 ± 5^*^
WM:cross-sectional area (mm^2^)	0.1 ± 0.01	0.1 ± 0.02	0.1 ± 0.01	0.1 ± 0.02	0.2 ± 0.02	0.2 ± 0.01
The number of Purkinje cells (mm)	0.016 ± 0.002	0.016 ± 0.001	0.015 ± 0.001	0.013 ± 0.001	0.015 ± 0.001	0.016 ± 0.001

Mean ± SEM are presented for each group.

* p ≤ 0.05 vs. control.

d GA, days gestational age; FGR, fetal growth restriction; EGL, external granular layer; ML, molecular layer; WM, white matter.

### Experimental Timeline

All animals were monitored daily from surgery until the day of postmortem. The ewe and fetuses were euthanased at three different ages to allow for comparisons of brain development and injury. At 115 and 124 d GA, animals were killed with maternal injection of pentobarbitone (Lethabarb; Virbac, Peakhurst, Australia) and the fetuses were removed, weighed, and brains were collected for analysis. In the third cohort, lambs were born naturally at term (~145 d GA) and at 24 h of age, lambs were euthanased with an intravenous injection of pentobarbitone; the lamb was weighed and brains were collected for analysis. The cerebellum of each animal was divided into right and left halves. The right half of the cerebellum was fixed by immersion in 4% buffered paraformaldehyde (PFA; ProSciTech, Thuringowa, QLD, Australia) for 3 days and transferred to 70% ethanol overnight, prior to embedding in paraffin. Subsequently, 10-μm coronal sections were cut for analyses.

### Immunohistochemistry and Histological Stains

Sections were dewaxed and rehydrated through graded ethanol transfer before commencing the procedures described below. All washes were carried out in phosphate-buffered saline (PBS, 0.1 M, pH 7.4). For each immunohistochemical protocol, duplicate sections of the cerebellum from all treatment groups were included in a single run to eliminate variations between runs.

Glial fibrillary acidic protein (GFAP) was used to visualize astrocytes and astrocytic end-feet. Ionized calcium binding adaptor molecule 1 (Iba-1) was used to identify microglia. The DNA adduct 8-hydroxy-2-deoxyguanosine (8-OHdG) was used as a marker of oxidative DNA damage. NEUronal nuclei, clone A60 (NeuN), was used to identify neuronal nuclei; microtubule-associated protein 2 (MAP-2) was used to examine neuronal filaments, and albumin was used to detect blood protein extravasation. GFAP, Iba-1, 8-OHdG, NeuN, MAP-2, and albumin were identified using the following: monoclonal mouse anti-GFAP (1:400; Sigma-Aldrich), rabbit anti-Iba1 (1:1,000; Wako Pure Chemical Industries Ltd., Osaka, Japan), mouse anti-NeuN (1:200; Millipore Corporation, USA), mouse anti-MAP-2 (1:200; Thermo Fisher Scientific, Waltham, Massachusetts, USA), and rabbit anti-sheep albumin (1:1,000; Accurate Chemical and Scientific Corp., Westbury, NY, USA), respectively. Antibodies were diluted in PBS. All sections were treated with a secondary antibody (1:200; biotinylated anti-rabbit or anti-mouse IgG antibody; Vector Laboratories, Burlingame, CA, USA) and immunolabeling was visualized using 3,3-diaminobenzidine (DAB; Pierce Biotechnology, Rockford, IL, USA). Positive and negative control sections were included in each run. Sections were viewed at 400× magnification using a light microscope (Olympus BX-41, Japan).

### Perls’ Prussian Blue

Perls’ staining was used to observe the presence of microbleeds (which presented as free iron) within the cerebellum. Following dewaxing, the slides were rehydrated in distilled water for 5 min. While this took place, a solution of equal parts 4% aqueous potassium ferrocyanide and 4% hydrochloric acid was mixed, and then heated to 60°C in a microwave that was placed within a fumehood to prevent exposure to hydrogen cyanide fumes. Slides were then placed into the heated solution for 40 min and then allowed to cool. The sections were rinsed in running distilled water for 2 min and then placed into a dish and counter-stained with Nuclear Fast Red for 4 min. Following this, the slides were rinsed for another 2 min under running distilled water, and then dehydrated in ascending alcohol concentrations, cleared in xylene, and cover-slipped. Sections were examined for the presence of microbleeds using a light microscope (Olympus BX-41, Japan). All areas of the cerebellum were examined, and bleeds were counted manually.

### Image Analysis

**Figure 1:**
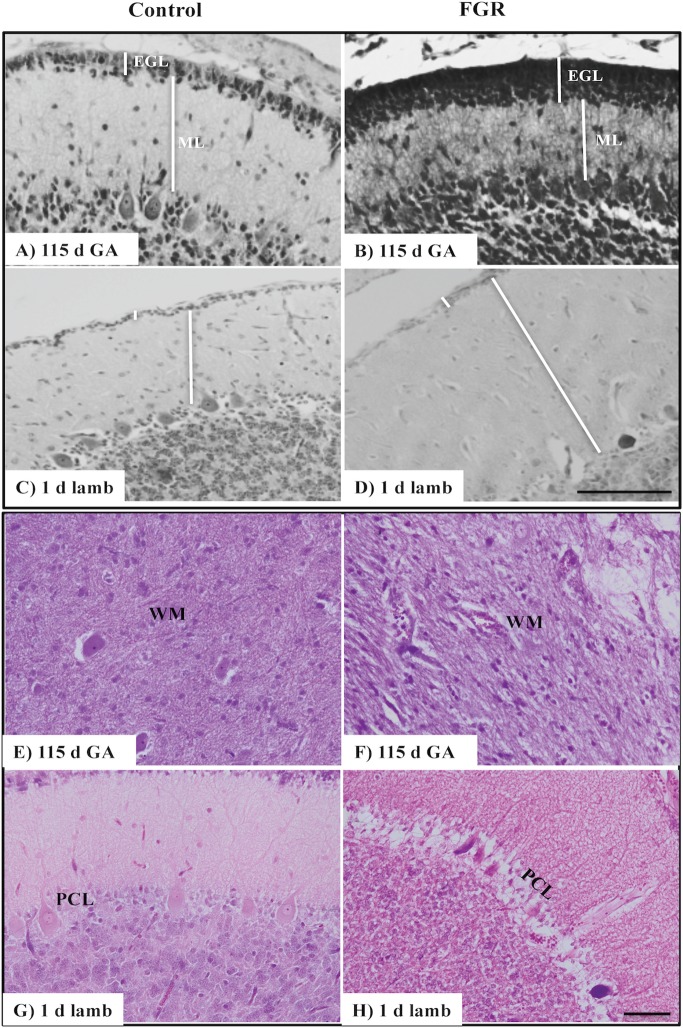
Representative micrographs of cresyl violet and acid fuchsin staining in a 115 d GA fetus and 1-day-old lamb of control **(A,C,E,G)** and FGR **(B,D,F,H)** brain. **(A–D)** show measurements of EGL and ML on each image. Within FGR brains, note the presence of vacuolization in parenchymal WM tissue **(F)** and Purkinje neurons with abnormally shaped, darkly stained nuclei **(H)**. EGL, external granular layer; ML, molecular layer; PCL, Purkinje cell layer; WM, white matter. Scale bar for **(A–D)** in **(D)** = 100 μm and **(E–H)** in **(H)** = 50 μm.

Densitometric calculations for GFAP, 8-OHdG (internal granular layer; IGL), and MAP-2-positive immunoreactivity was performed using a computerized image analysis system (ImageJ version 10.2, NIH, Bethesda, Maryland, USA) that reads optical density as gray levels, at 400× magnification. An investigator (TY), blinded to the experimental groups, selected the threshold levels of detection, where nonspecific background was identified and the threshold level was then kept constant for all images within a given immunohistochemistry run. For cell counts and scoring, two sections of the cerebellum per animal were examined, and the number of immunopositive cells for 8-OHdG [molecular layer (ML) and WM] and NeuN, or percentage area occupied by Iba-1-positive cells, was calculated using an average of three fields of view per area, and the results were averaged across the animals in each group. Analysis for cresyl violet and acid fuchsin staining, Purkinje cells, IGL and ML width, GFAP, 8-OHdG, Iba-1, MAP-2, and NeuN immunohistochemistry was performed in the anterior and posterior lobules (II, VI, and VIII), in three randomly selected fields of view. Analysis for microbleeds and albumin extravasation was performed on the entire section of the cerebellum, including all folia. The number of incidences for microbleeds and albumin extravasation was counted and averaged per section in duplicate across all animals in each group. Neuronal cell morphology was assessed using cresyl violet and acid fuchsin staining; cells that displayed swelling of organelles, loss of cell wall, and/or nuclear membrane integrity were considered abnormal. The cross-sectional area of the entire cerebellum and the WM were performed on the cresyl violet and acid fuchsin-stained sections using Image J to trace around each area, and data for cerebellar WM are presented per total area. The IGL and ML width were measured using the same program and data presented as μm (Figure 1).

### Data Analysis

Data are presented as the mean ± standard error of the mean (SEM). Statistical analysis was performed with GraphPad Prism 7 (GraphPad Software, San Diego, United States). An unpaired nonparametric *t*-test was performed to compare between the control and FGR group at each gestational age. Significance was accepted when *p* ≤ 0.05.

## Results

We induced late-onset placental insufficiency in fetal sheep (refer to Table 1 for *n* numbers), which resulted in chronic fetal hypoxia that worsened compared to control fetuses over the course of gestation; 115 d GA 52 vs. 59% FaO_2_, 124 d 58 vs. 70%, and 142 d GA 43 vs. 62% FaO_2_ (Table 1). The success rate for fetal survival after FGR surgery was 83%, with all fetal deaths occurring within the first 24 h post-surgery. Body weights were reduced in FGR fetuses compared to age-matched controls, and this was statistically significant at 124 d GA and in 1-day-old lambs (Table 1). Brain weight was spared in FGR fetuses and lambs, such that the brain to body weight ratio was significantly increased at 124 d GA and in 1-day-old FGR lambs. The total area of the cerebellum to body weight ratio was also significantly increased in the FGR 1-day-old lambs compared to controls. FGR had no effect on the area of cerebellar WM (Table 1).

### Morphological Changes in the Cerebellum

We first assessed basic cerebellar morphology in the control animals. Cresyl violet and acid fuchsin staining showed that, at 115 d GA, a prominent external granular cell layer (EGL) was apparent in control brains (Table 1), which actively proliferates over the preterm period studied, wherein the cells migrate inward to form the IGL (Volpe, 2009). After 124 d GA, the EGL was less prominent, and was further decreased in width in 1-day-old control lambs (EGL at 115 d: GA 22.0 ± 1.6 μm, at 124 d: GA 21.7 ± 2.3 μm, and in 1-day-old lambs: 14.1 ± 0.7 μm). By contrast, the width of the ML in control animals increased as gestation progressed (Table 1). At 115 d GA, the width of the EGL in FGR fetuses was significantly thicker than control fetuses (Figures 1A,B), but this difference was no longer seen at 124 d GA and in 1-day-old lambs. In FGR animals compared to control animals, the width of the ML was significantly reduced at 115 d GA; no difference was observed at 124 d GA, but then became significantly wider in 1-day-old lambs (Figures 1C,D).

At 115 d GA, Purkinje cell bodies were evident in control brains, and the number of Purkinje cells remained relatively consistent from 115 d GA through to 1-day-old postnatal lambs (Table 1). The IGL had a dense population of neurons, and the boundaries between IGL and the WM were also apparent at 115 d GA. Qualitative analysis revealed that the population of cells in the IGL appeared to be increased by 124 d GA and even further in 1-day-old lambs, and sharp boundaries were formed between the IGL and WM, such that the two layers were highly distinguishable.

There was no evidence of gross lesions within the FGR brains, but at 115 d GA, FGR fetuses showed evidence of neurons with abnormally shaped, darkly stained nuclei, vacuolization, and patchy coverage in parenchymal WM tissue (Figure 1F); this was also evident in FGR fetuses at 124 d GA and in 1-day-old lambs. We noted that the cerebellar Purkinje cells exhibited altered cellular morphology and arrangement in FGR brains, as evidenced by intensely stained Purkinje cell bodies of abnormal shape and Purkinje cell disorganization, most apparent in 1-day-old lambs (Figure 1H).

**Figure 2:**
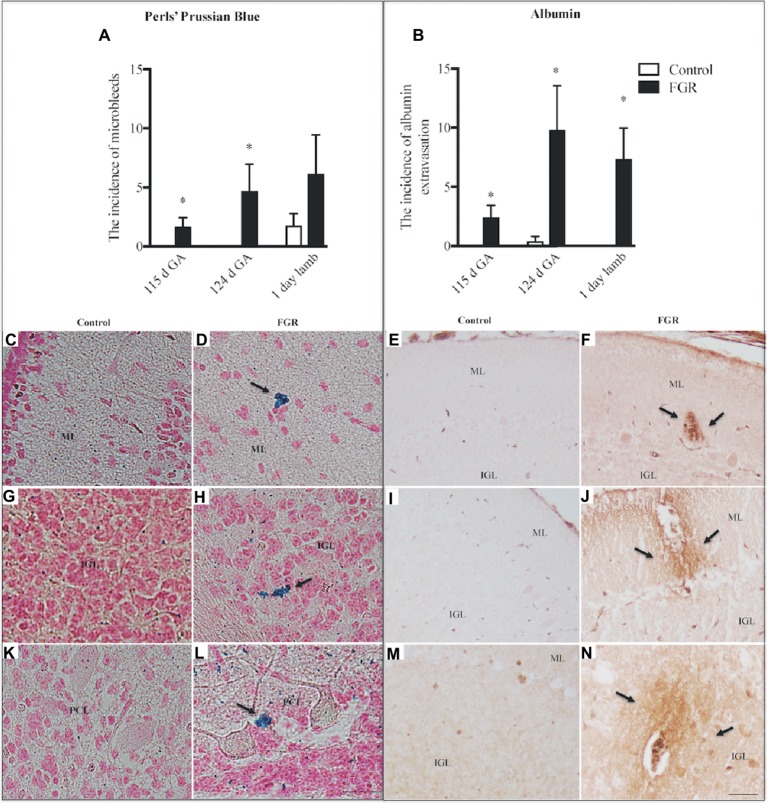
The number of microbleeds **(A)** and albumin extravasation from blood vessels (BVs) **(B)** in the cerebellum of the fetal brain at 115 d GA, 124 d GA, and 1-day-old lamb of control and FGR animals. Each bar represents the mean ± SEM; *p ≤ 0.05 versus control. Representative micrographs showing Perls’ Prussian Blue staining and albumin immunohistochemistry in a 115 d GA fetus **(C–F)**, 125 d GA **(G–J)**, and 1–day-old lamb **(K–N)** brains. Microbleeds were visualized as bright blue patches that were counter-stained with nuclear fast red. Arrows indicate the sites of microbleeds/albumin extravasation. FGR, fetal growth restriction; PCL, Purkinje cell layer; ML, molecular layer; IGL, internal granular layer. Scale bar for Perls in **(L)** = 25 μm. Scale bar for albumin in **(N)** = 50 μm.

We investigated the presence of microbleeds within the cerebellar tissue (Figure 2A), visualized as bright blue patches (Perls’ positive staining), counter-stained with nuclear fast red (Figures 2D,H,L). Control fetuses did not exhibit any blue staining; (Figures 2C,G,K) however, microbleeds were present in 4/8 control lambs. In FGR brains, blue staining was present in 4/6 fetuses at 115 d GA, and 5/5 fetuses at 124 d GA, with microbleeds present within the ML, IGL, and WM. Microbleeds were also identified in 5/7 FGR lambs.

The absence of immunoreactive albumin within the brain is indicative of a functional BBB, and albumin was not observed within control fetal and lamb brains (Figures 2B,E,I,M), with the exception of albumin extravasation in one of five control animals at 124 d GA. In FGR brains, the occurrence of albumin extravasation from blood vessels (BVs) was observed in all regions of the cerebellum (Figure 2B). Where albumin was present in FGR brains, intense immunoreactivity was predominantly observed in brain parenchyma adjacent to BVs (Figures 2F,J,N).

### Immunohistochemistry

**Figure 3:**
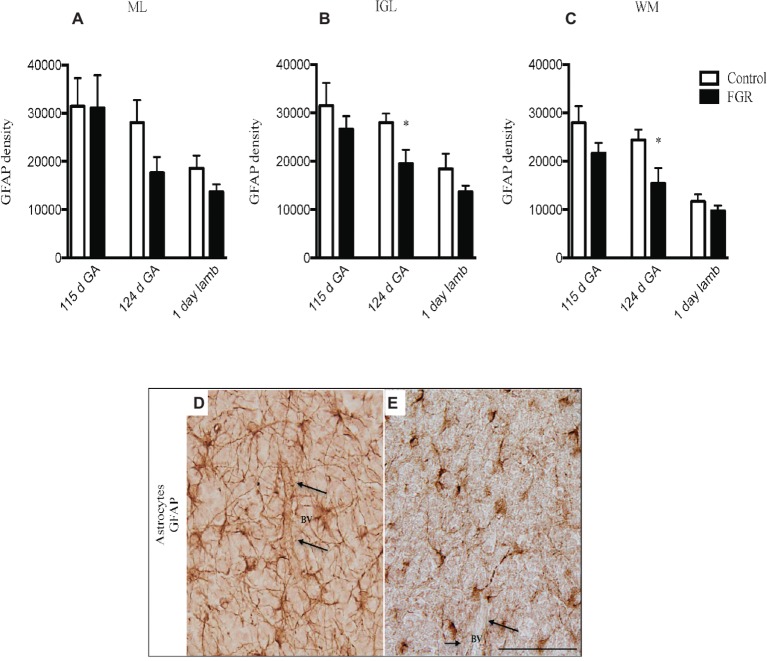
Glial fibrillary acidic protein (GFAP) density in the molecular layer [ML **(A)**], internal granular layer [IGL **(B)**], and white matter [WM **(C)**] in the fetal brain at 115 d GA, 124 d GA, and 1-day-old lamb of control and FGR animals. Comparisons are made within the same brain region at the same gestation and not across regions or across gestation. Each bar represents the mean ± SEM; *p ≤ 0.05. Representative micrographs showing GFAP-positive immunoreactivity in the fetal IGL at 124 d GA of control **(D)** and FGR **(E)** brain. Arrows pointing to a blood vessel (BV), showing the damaged astrocytic end-feet. FGR, fetal growth restriction. Scale bar = 50 μm.

GFAP-positive astrocytes were present in the cerebellar ML, GL, and the WM of control and FGR brain. At 115 d GA, astrocytes were not affected by FGR. However, the density of the astrocytic cell bodies and processes appeared to be reduced in all FGR brains at 124 d GA and 1-day-old lambs compared to control animals but this was only statistically significant in the IGL and WM at 124 d GA (Figures 3A–C). Furthermore, we observed very few end-feet of astrocytes associated with BVs in FGR animals (Figures 3D,E). The association of astrocytic end-feet to BVs is critical for maintenance of the BBB, and therefore, in this situation, a decrease in astrocyte density and end-feet attachment has likely compromised the BBB in FGR offspring.

**Figure 4:**
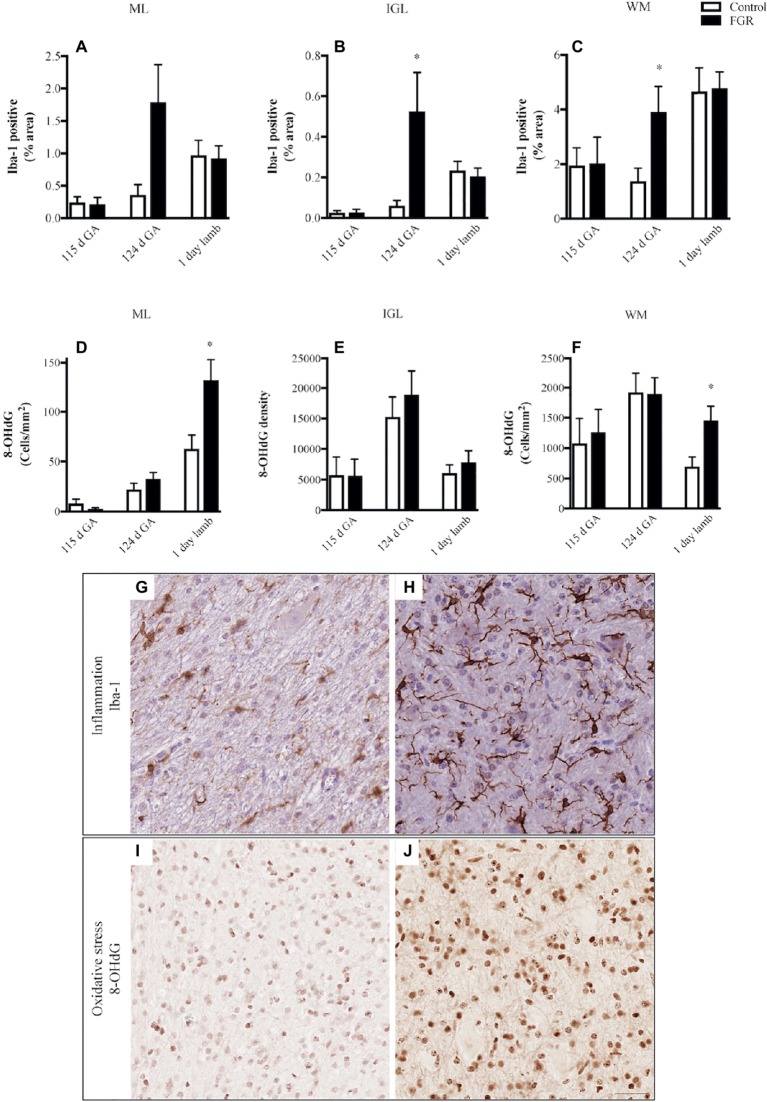
The number of Iba-1-positive cells **(A–C)** and 8-hydroxy-2-deoxyguanosine (8-OHdG)-positive cells **(D–F)** in the molecular layer (ML), internal granular layer (IGL), and white matter (WM) in the fetal brain at 115 d GA, 124 d GA, and 1-day-old lamb of control and FGR animals. Each bar represents the mean ± SEM; *p ≤ 0.05 versus control. Representative micrographs showing Iba-1- **(G,H)** and 8-OHdG- **(I,J)** positive immunoreactivity in the WM in 124 d GA **(G,H)** and 1-day-old lambs **(I,J)**. Images from control **(G,I)** and FGR **(H,J)** animals are shown. FGR, fetal growth restriction. Scale bar = 25 μm.

We quantified microglial cells, and found that in FGR fetuses at 124 d GA, there was an overall increase in the percentage area coverage of microglia that was significant in the GL and WM (Figures 4A–C). There was no difference in microglial cell coverage in FGR (Figure 4H) and control (Figure 4G) animals at 115 d GA and 1-day-old lambs.

We next examined whether oxidative stress was upregulated in the FGR cerebellum. Cells that were positive for 8-OHdG immunoreactivity were present in control and FGR brains (Figures 4I,J). The number of cells that were positive for 8-OHdG was significantly increased in FGR 1-day-old lambs within the ML and WM compared to control brains (Figures 4D–F).

**Figure 5:**
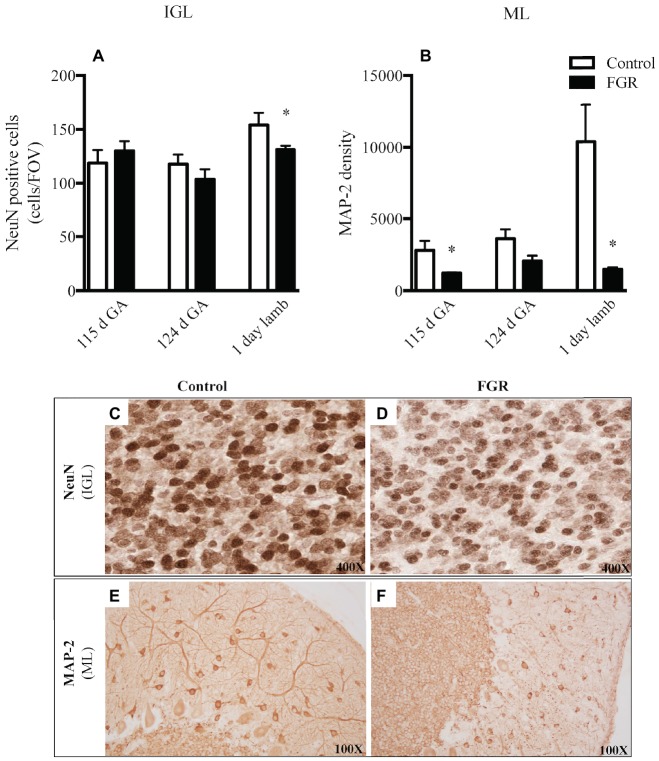
The number of NeuN-positive cells per field of view **(A)** and MAP-2 density **(B)** in the internal granular layer (IGL) and molecular layer (ML) in the fetal brain at 115 d GA, 124 d GA, and 1-day-old lamb of control and FGR animals. Each bar represents the mean ± SEM; *p ≤ 0.05 versus control. Representative micrographs showing NeuN-positive immunoreactivity in the IGL **(C,D)** and MAP-2 in the ML **(E,F)** in 1-day-old lambs. Images from control **(C,E)** and FGR **(D,F)** animals are shown. FGR, fetal growth restriction.

We examined neuronal cell number (NeuN) and the morphology of neuronal filaments (MAP-2) within the cerebellum. We utilized NeuN as a neuronal-specific nuclear protein (Mullen et al., 1992) and, as expected, NeuN-positive cells were localized to the cerebellar IGL and not the ML and WM, which is consistent with published data (Zimatkin and Karniushko, 2016), indicative that we had appropriately labeled for mature IGL neurons. The number of mature neurons was significantly reduced in FGR brains of 1-day-old lambs within the IGL compared to control lambs (Figures 5A,C,D). When we examined the expression of the MAP-2-positive immunoreactivity within the ML, a region where neuronal filaments extend to other areas in the cerebellum, we observed an overall decrease in the expression of MAP-2 at all gestational ages in the FGR animals, but this decrease was statistically significant at 115 d GA and 1-day-old lambs, compared to control (Figures 5B,E,F).

## Discussion

Here, we examined the effects of placental insufficiency, chronic fetal hypoxia, and FGR on cerebellar development at three timepoints corresponding to the last trimester of human pregnancy and into the neonatal period. We show that cerebellar growth and development are adversely affected soon after the onset of hypoxia, and that chronic fetal hypoxia leads to a progressively altered developmental profile of the cerebellum. This is the first study to characterize maturation of normal and abnormal morphology within cerebellum from appropriately grown and growth-restricted fetuses and neonates. Most strikingly, newborn FGR lambs demonstrated neuropathology within the cerebellum after birth, with a significant, ~18% decrease, in the number of granule cell bodies within the IGL and an ~80% reduction in neuronal extension and branching within the ML. The foundations of neonatal cerebellar neuropathology were laid down soon after the onset of placental insufficiency and fetal hypoxia. The structural integrity of neurons was already aberrant in the FGR cerebellum at 115 d GA, and by 124 d GA, inflammatory cells were upregulated and the BBB was compromised. These neuropathologies occurred in the absence of gross cerebellar abnormalities; the cross-sectional area of the whole cerebellum and the WM were not different in size compared to control, and no cystic lesions were present in the brain of FGR offspring. Thus, we show that chronic *in utero* compromise leads to progressive maturational deficits in the cerebellum of FGR offspring, likely to have significant functional implications.

We induced late-onset placental insufficiency to mimic the clinical situation of FGR that is most commonly observed (Baschat, 2014). This ovine model of late-onset FGR did not significantly affect body weight 10 days after induction of placental insufficiency (115 d GA); however, body weight was significantly reduced in FGR offspring at 19 days (124 d GA) and 43 days (1-day-old lamb) following the onset of placental dysfunction. This was accompanied by a significant increase in brain to body weight ratio, a finding that supports our previous work (Miller et al., 2009, 2014) and reflects asymmetric growth in response to chronic hypoxia (Baschat, 2014; Miller et al., 2016).

It was interesting to note that, while body weight was not yet affected at 115 d GA, FGR fetuses were already experiencing significant hypoxia, and this was associated with early signs of cerebellar neuropathology. At 115 d GA, many cerebellar granule cells in FGR animals were abnormally shaped, with darkly stained nuclei – not observed in control fetuses – and similar cellular abnormalities remained evident at 124 d GA and in 1-day-old lambs. This supports previous work showing that both acute and chronic hypoxia result in cerebellar neuropathology, as described in fetal sheep (Yawno et al., 2009), rats (McDougall et al., 2017), and guinea pigs (Tolcos et al., 2018); however, these studies did not examine the ontogeny of the cerebellum. Structurally, at 115 d GA, the width of the EGL in FGR animals was significantly increased, and this was associated with a significant decrease in the width of the ML compared to control animals. The changes in the EGL and ML were resolved at 124 d GA. However, in 1-day-old FGR lambs, the width of the ML was significantly increased compared to control lambs. These findings support previous results from other models of FGR (McDougall et al., 2017; Tolcos et al., 2018), where it was suggested that the increase in EGL width maybe due to impaired granule cell migration out of the EGL, increased cell proliferation, and reduced granule cell apoptosis. Even if these structural changes were corrected at 124 d GA, alterations were still evident in 1 day-old FGR lambs, which could lead to changes in cellular connectivity and cerebellar circuitry. Hence, further investigations on cell migration, proliferation, and apoptosis are warranted.

We noted that Purkinje cell bodies were morphologically abnormal, and the Purkinje cell layer was highly disorganized in 1-day-old FGR lambs only, thereby suggesting that progression of neuropathology over the course of late gestation had a direct effect on the Purkinje cell layer. Reduction in Purkinje cell number has been reported in the cerebellum of growth restricted guinea pigs and was associated with neurological and behavioral deficits postnatally (Mallard et al., 2000). While we did not see a change in the total number of Purkinje cell in our FGR offspring, it is known that Purkinje cells receive excitatory inputs from injured granule cells within the IGL (Sarna and Hawkes, 2003), thereby potentially making them more susceptible to abnormal development and injury.

A notable observation in the current study was the susceptibility of the cerebellum of FGR animals to hemorrhage. More than 80% of FGR fetuses (both 115 d and 124 d) showed evidence of brain bleeds, compared to no bleeds within control fetuses. In addition, we observed the presence of immunoreactive albumin adjacent to BVs within the brains of all FGR animals (115 d, 124 d, and 1-day-old lambs). This result is likely due to altered cerebrovascular development and suboptimal integrity of the BBB. We have previously examined cerebrovascular development within the subcortical and periventricular WM of the cerebrum, and shown specific cerebrovascular deficits within the FGR brain that include decreased basal lamina area, reduced vascular endothelial growth factor (VEGF), fewer endothelial cells, and reduced BBB integrity (Castillo-Melendez et al., 2015, 2017). The structural and functional features of the BBB are normally maintained by the contribution and cellular interactions of the endothelial cells, pericytes, and astrocytic end-feet contacts (Kim et al., 2013). In particular, close astrocyte association with BVs is important in development as it provides functional and structural support by controlling inflammatory cell infiltration and maintaining homeostasis (Farhy-Tselnicker and Allen, 2018). Thus, our finding that the FGR brain showed reduced expression of astrocytic density from 124 d GA potentially plays a critical role in abnormal brain development. In addition, with reduced glial support, neuronal function may be even further compromised (Laming et al., 2000). It is worth noting that the presence of microbleeds (Perls’ stain) in our control lamb brains was unexpected; we are unsure to why this has happened; however, all lambs in this cohort were delivered naturally which could induce temporary instability of the BBB *via* hypoxia at birth, but it is also important to note that the albumin data show that control lambs did not show any albumin extravasation and an indication of a more stable BBB.

Concomitantly with altered BBB function at 124 d GA, we also saw a neuroinflammatory response within the cerebellum of FGR fetuses in this age group, with a large increase in microglial cells in all cerebellar regions examined, compared to control brains. This finding fits with growing knowledge of the contribution of neuroinflammation toward neuropathology in FGR animal studies (Wixey et al., 2018) and in human preterm infants (Buser et al., 2010). Inflammation-induced brain injury is associated with WM damage, acute BBB dysfunction, and neurovascular unit injury, and in turn linked with behavioral alterations (Stolp et al., 2011). It is interesting to note that an elevation in neuroinflammation was only observed within the FGR brain at 124 d GA, and this was linked with an altered astrocyte profile at this age, and high susceptibility to hemorrhage. Previous work has shown that injured glial cells release factors that act on target cells to initiate responses of activated immune cells in the periphery, in turn leading to inflammatory cell infiltration of tissues. These events usually occur when there has been destruction or compromise of the BBB (Streit et al., 1998; Sroga et al., 2003). Taken together, these results are indicative that this developmental timepoint (roughly equivalent to 34 weeks in human cerebellar development) is particularly vulnerable for BBB dysfunction linked with upregulation of neuroinflammation. It is of interest to note that clinically, rates of intraventricular hemorrhage (IVH) are not increased in FGR infants born preterm at 28 weeks compared to appropriately grown infants, but IVH rates are significantly elevated in human infants born late preterm (>34 weeks) (Gilbert and Danielsen, 2003; Ortigosa Rocha et al., 2010), indicative of the vulnerability of the BBB in late gestation (Wixey et al., 2018). It is, however, worth noting that the marker we used to identify inflammatory cells does not discriminate between resting and activated microglia. Therefore, it would be of interest to further phenotype the inflammatory cells to examine whether late-onset FGR induces a pro-inflammatory environment within the cerebellum, which may be amenable to treatment.

We examined the presence of oxidative DNA damage (8-OHdG) to indicate whether oxidative stress was upregulated in the cerebellum of FGR animals and contributed to the neuropathology observed (Yawno et al., 2017). During fetal gestation, both control and FGR animals showed cellular oxidative stress to be present within the cerebellum, with no differences seen between the groups. In 1-day-old lambs, oxidative cell damage was significantly upregulated within the cerebellar ML and WM of FGR offspring, compared to control animals. All lambs were born naturally at term and cared for by their mother after birth, with no need for intervention or supplemental oxygen. Therefore, the observation of increased oxidative damage within the brain of newborn FGR lambs may represent a response to birth. Potentially, this is contributed by relative hyperoxia experienced by FGR lambs after birth compared to control lambs, given their *in utero* conditions of chronic hypoxia. It may also be due to the reduced anti-oxidant defense capabilities in the FGR lamb. We have previously shown that in FGR offspring, circulating malondialdehyde concentration (a marker of oxidative stress) was elevated compared to appropriately grown offspring (Polglase et al., 2017) and that the same FGR offspring had significantly increased levels of oxidative stress within the brain, in both neuronal cell bodies of the cortex and fiber tracts within the periventricular WM (Miller et al., 2014). This is the first study to show that oxidative stress is upregulated in the FGR cerebellum, appearing to affect granule cell axons and migrating neurons within the ML. Oxidative DNA damage might be a precursor to cell death, which would require substantiation in long-term studies.

Mature IGL neurons (NeuN-positive cells) were significantly reduced in the neonatal FGR brains compared to control lambs but no difference was apparent at earlier timepoints, thereby suggesting that cerebellar neuropathology resulting from late-onset FGR takes time to manifest. This finding is in agreement with other literature showing a reduction in the density of cells undergoing mitosis in the cerebellar granular layer of hypoxemic fetal sheep compared to controls (Rees et al., 1997) and suggests that chronic hypoxia is causing an arrested maturational profile in IGL neurons or that mature neurons have not completed migration into the IGL. In addition to reduced total number of neurons in the granular layer, we also observed a decrease in the expression of MAP-2 across gestation in the ML of all FGR animals, indicative of disrupted dendritic branching. MAP-2 is critical for neurite extension and branching, and for cessation of cell division (Johnson and Jope, 1992). In rodents, it has been shown that loss of MAP-2 expression is associated with neuronal degeneration, which has short- (Kinugasa-Taniguchi et al., 2010) and long-term (Matesic and Lin, 1994) effects on cognition and anxiety-related behavior (Matesic and Lin, 1994). Fundamentally, loss of neurons and altered neuronal morphology of existing neurons may well underlie deficits in spatial motor function and coordination, which are present in children who were growth restricted at birth (Tolsa et al., 2004).

A noted limitation of the current study is that we did not have sufficient numbers of animals to examine potential sex differences. A recent review has described important female versus male differences in fetal growth and sex-specific response to preterm birth and compromised placental function (Alur, 2019). In further studies, it would be of interest to examine cerebral and cerebellar development in males and females following compromised intrauterine environment, with the potential for tailored therapeutics.

Cerebellar injuries are often overlooked in experimental and clinical studies of brain neuropathology associated with acute or chronic hypoxia. Here, we demonstrate the progressive nature of developmental deficits in the cerebellum, associated with increasing placental insufficiency, chronic hypoxia, and FGR. These results suggest that the period of suboptimal *in utero* environment contributes to cerebellar pathology in late gestation fetal sheep. These results also indicate that late preterm age in sheep is a time of high risk for glial disturbances. Glial cells play an important function in the survival and maintenance of the neurovascular unit, and conversely, altered glial number or function can cause disruption to the BBB and brain developmental more broadly. We confirm that cerebellar injuries develop antenatally in FGR, and therefore, interventions to prevent long-term motor and coordination deficits should be implemented antenatally and/or perinatally, potentially targeting neuroinflammatory and oxidative stress pathways.

## Ethics Statement

This study was carried out in accordance with the recommendations of Monash Medical Centre Animal Ethics Committee A. The protocol was approved by the Monash Medical Centre Animal Ethics Committee A.

## Author Contributions

TY performed the experiments and data analysis, and wrote the original draft of the manuscript. GJ, MC-M, and SM provided funding, designed the experimental protocol, and helped draft the manuscript. AS assisted with animal experiments and data analysis, and helped draft the manuscript. YP assisted with data analysis.

### Conflict of Interest Statement

The authors declare that the research was conducted in the absence of any commercial or financial relationships that could be construed as a potential conflict of interest.
